# Gastrojejunostomy; with Report of a Case and Presentation of Specimen1Read before the Syracuse Academy of Medicine, October 31, 1905.

**Published:** 1906-03

**Authors:** Aaron B. Miller

**Affiliations:** Syracuse, N. Y.


// BUFFALO MEDICAL JOURNAL.

VOL. LXI. MARCH, 1906. No. 8

### ORIGINAL COMMUNICATIONS.

### Gastrojejunostomy; With Report of a Case and Presentation of Specimen[1].

By AARON B. MILLER, M. D., Syracuse, N. Y.

THIS paper is based on the clinical history of a case which is summarised as follows: Mrs. D, housewife, aged 36 years, consulted me at my office in January, 1904. She was the mother of three children, the oldest 17; the youngest 12. For six years she had suffered from attacks of indigestion, and of late was having continuous pain in stomach which was increased by the use of solid foods. She had been told that her condition was due to pelvic trouble and it was for this reason she came to consult me.

My examination evolved the following data: monthly sickness regular; number of days 4 and napkins 6 to 10. While she had suffered pain in the early menstrual history, it had disappeared after child-birth. She complained of a sensation of weight in the lower abdomen; sacralgia, constipation and leucorrhea. An examination showed the pelvic floor broken down, with rectocele and vaginocle present; the cervix had been torn, the uterus was subinvoluted and was attended by hypertrophy and hyperplasia.

The stomach symptoms seemed to be aggravated by the menstrual period, but at no time did they abate, and were increasing in severity from month to month. Examination of the abdomen elicited general soreness, more marked in the region of the stomach, and a fixed, rigid abdominal wall. Physical examination of the abdominal organs was negative; local treatment was advised to be continued for the pelvic trouble and medication ordered for the stomach symptoms.

In June, 1904, I was called to see her at her home in consultation with the family physician. I found her in bed, weak and pale, expression anxious and sallow. A few days before she had vomited a quantity of blood and the stools now were tarry

---

1 Read before the Syracuse Academy of Medicine, October 31, 1905.

in appearance. Emaciation was marked and vomiting had been frequent for two weeks; all solid foods had been stopped and fluids were not retained at all times. Tenderness was more marked over the pyloric extremity of the stomach; gallstones were thought of but the history never suggested liver disturbance. Upon questioning her more carefully she then stated that solid food had not been retained for four months and that frequently the ejecta contained blood. I advised she should enter the hospital and the condition should be treated surgically, hoping it might prove to be a benign thickening at the pylorus induced by ulceration, stating that if it should prove to be carcinomatous her chances for relief could not be lessened by such proceedure.

Further counsel was called subsequent to my visit; a diagnosis of cancer was made and the patient advised against operation. This I learned upon being asked to see her again in July. At this time I was still unable to determine a tumor, though the patient said it was present at times. Emaciation had progressed, liquid being the only food taken; vomiting increasing in frepuency and pain intensified. I again advised operation and was told it would be considered. I failed to hear further from the patient until after my summer vacation, when I learned she was receiving care from the so-called "Christian scientists" and hoped for relief without submitting to an operation.

In the early part of October I was summoned again; the patient had just had a severe hemorrhage and estimated the amount of the emesis at 3 pints; she was very much exhausted but begged something might be done. Saline enemas with nutriment were ordered and anodynes given to relieve pain. Her appearance was that of the India famine sufferers, as pictured. The anterior abdominal walls had assumed the characteristic shortening and was so attenuated that the vermicular action of the intestines could be studied through them. At this time, while inspecting the abdomen, the tumor appeared, induced by the contraction of the muscular walls of the stomach and was located to the left of the median line, the ulcer in the pyloric or duodenal end acting as the excitant. The patient was conscious of its presence, exclaiming "now there is a ball in my stomach." This soon passed away and with the rigidity of the abdominal walls it would have been impossible to have appreciated any enlargement beneath by palpation. It was decided if she continued to improve that she should be taken on a cot to the hospital in a few days.

October tenth, five days after my visit, she entered the hospital having been brought a distance of fourteen miles on a bed, without suffering any ill effects excepting fatigue, from which she soon rallied; pulse, temperature and respiration slightly accelerated, but not of marked pathologic significance. Preparations were commenced at once for surgical intervention; concentrated liquid foods were ordered and efforts made to improve her general condition, while anticipating another hemorrhage.

Sufficient codeine to alleviate pain was given. In observing the stomach from time to time it was of the greatest interest to notice it contract into a hard ball, this to relax or pass away and return at irregular intervals. The ninth day after entering the hospital she was operated upon; the incision was made through the right rectus, the stomach examined and found free from involvment, except at the pyloric end, which was hard and its lumen nearly closed. I need not describe the technic of the operation. The von Hecker method was resorted to, that is, a posterior anastomosis was made, joining the jejunum to the most dependent portion of the stomach wall. I was impressed with the ease and rapidity with which it was accomplished. In less than 45 minutes from the time the abdomen was incised, the patient was removed from the operating table, and it is my belief that the time could be reduced to 20 minutes in a similar case.

I lay emphasis upon this observation as I am convinced that in a patient reduced as this one was by prolonged anesthetisation and a tedious operation, shock would frustrate the recovery. Moreover, I would apply this rule to all operations in the abdominal or pelvic cavities, and especially where the intestines are concerned in the procedure. Upon the return of the patient to her room, transfusion was ordered and two pints of normal saline was given intravenously, followed by stimulating enemata every five hours for two days to bathe the tissues, relieve thirst and increase the blood pressure.

She vomited once, the latter part of the second day, the only time subsequent to the operation; this might have been due to the anesthetic or to a small amount of anodyne given when she was taken from the operating room, to relieve restlesseness while recovering from anesthesia. After 24 hours. sips of hot water were given by the mouth, gradually followed by liquid nourishment consisting of beef tea, milk, gruel and cereals, until the eleventh day, when solid foods consisting of chicken, graham crackers, and the like, were ordered. On the nineteenth day after the operation she was permitted to sit up in a chair and three days afterward left the hospital, walking to the carriage. Less than five weeks after the operation I called upon her to inquire if she was comfortable and learned that she was free from pain or distress, but owing to her hunger was ignoring all dietary laws, even getting up in the night to eat cold baked beans.

During the spring of the present year, Mrs. D. called at my office; she had been comfortable during the winter and had gained thirty pounds in weight since her return home. At no time had she suffered from the food she took, had been able to do her house work, and had been with her daughter in her first confinement. In July she called again, complaining of a feeling of weight in her right side; examination showed the liver to be enlarged. I saw her again August 28; she had been having a dull pain in the liver region; facial expression not as good; menses stopped May 17; liver found to be much enlarged, extending one-half way down to the ilium and filling the abdomen to the median line. While there were no stomach symptoms, her general appearance was bad; she was rapidly losing weight and was emaciated.

By September 10, the patient had grown markedly worse since her visit of August 28; she was confined to the bed and suffering from abdominal pressure; slept only with anodynes and took but little food. During the evening of Septmber 25, she cried out suddenly, saying that she had "split open;" examination revealed a quantity of discharge on the clothing which had come from the vagina. This was followed by intermittent pains. The family doctor was called and the following morning, at 4 o,clock, he relieved her of a four and a-half months fetus. October 7 she died, one year and three days after the operation.

---

A postmortem was granted at which the specimen which I present was secured. The emaciation was general and marked; abdomen protuding and large, liver filling greater part of abdominal cavity; weight after removal 13 lbs. The duodenum, pyloric end of stomach, and head of pancreas infiltrated and fixed; the specimen shows some pancreatic tissue. The stomach was comparatively free from involvment except locally at the pyloric end. Transverse colon free. Greater omentum entire and uninvolved.

Jejunum anastomosed to stomach. Attachment of mesocolon at point of junction. There were some small nodules in the mesentery, probably malignant; gallbladder free from involvment; intestines unembarrassed by disease. Pelvic organs free from disease; enlargement of the uterus from the impregnation; kidneys normal; liver examined by Dr. Stensland, pathologist, and pronounced carcinomatous.

---

While the case is not unsual, I present it because it has been an object lesson to me, and further because I was impressed with certain conditions that are common in many cases, which may be classified as follows.

I. The long continuance of symptoms and the probability that benign ulceration in the pyloric end of the stomach resulted in the subsquent malignancy. Possibly there might have been gallstones at the commencement, but the history does not aid us in this belief.

II. The contraction of the stomach into a hard ball, reflex from the ulceration, was perceptable to the touch and subsequently to the eye when the abdominal walls became attenuated and was located to the left of the median line or in the cardiac end.

III. The ease with which such an operation can be done after the technic is understood.

IV. The rapid and complete relief of symptoms, general improvement and prolongation of life.

V. The slight amount of stomach involvment as compared to the liver at postmortem.

VI. Pregnancy occuring under such conditions and for so long a period after the birth of the last child would scarcely have been looked for. Did pregnancy hasten the malignancy?

If I have been somewhat prolix in describing this case I have been stimulated to do so by foreseeing the possibilities, in intractible stomach troubles, in which resort to early operation would cure benign cases, afford immediate relief and prolong life in malignancy.

In conclusion, the expression of gratitude offered by the patient for the respite, and the comfort and happiness which she enjoyed during her last year would persuade me to urge more care in the diagnosis of stomach troubles and, when remedies fail to relieve and suspicion points to threatening conditions, as ulceration or malignancy, encourage the opinion that relief if not cure can be afforded by surgical methods.

326 MONTGOMERY STREET.

